# Case Report: Diabetic ketosis-associated hemichorea-hemiballism in an elderly patient with longstanding poorly controlled type 2 diabetes mellitus

**DOI:** 10.3389/fendo.2026.1991954

**Published:** 2026-10-26

**Authors:** Wennan Zhang, Lili Xu, Xinzhi Fu, Fengyao Sun, Yangang Wang

**Affiliations:** Department of Endocrinology and Metabolism, The Affiliated Hospital of Qingdao University, Qingdao, China

**Keywords:** case report, diabetic ketosis, diabetic striatopathy, hemichorea-hemiballism, type 2 diabetes mellitus

## Abstract

Hemichorea-hemiballism associated with ketotic hyperglycemia is a rare movement disorder. We report a case of right-sided hemichorea-hemiballism occurring in the setting of diabetic ketosis in a 70-year-old man with a 15-year history of poorly controlled type 2 diabetes mellitus. The patient presented with subacute involuntary choreiform movements of the right limbs, accompanied by foot pain, significant weight loss, and multiple diabetic complications. Intensive insulin therapy combined with short-term symptomatic treatment resulted in rapid resolution of ketonuria, stabilization of blood glucose, and marked improvement of the movement disorder. Notably, brain MRI showed no classic striatal T1 hyperintensity. At the 3-month follow-up, the patient remained free of choreiform movements. This case highlights that diabetic ketosis can trigger hemichorea-hemiballism in elderly patients even in the absence of typical imaging findings, and underscores the central role of prompt glycemic optimization

## 1 Introduction

Hemichorea-hemiballism (HCHB) secondary to hyperglycemia, commonly termed diabetic striatopathy (DS), is a rare but potentially reversible movement disorder (1). Although the majority of reported cases occur in the setting of non-ketotic hyperglycemia, a growing number of observations confirm that DS can also develop in the presence of ketosis or frank diabetic ketoacidosis (2–5). The precise pathophysiology remains incompletely understood and may involve striatal ischemia, petechial hemorrhage, metabolic dysfunction of GABAergic neurons, or hyperviscosity-related changes (6).

We report an elderly man with longstanding, extremely poorly controlled type 2 diabetes mellitus who developed right-predominant HCHB in the setting of diabetic ketosis. The case is notable for the combination of ketosis, advanced age, male sex, extensive diabetic complications, and the absence of classic striatal imaging abnormalities, yet it demonstrated an excellent clinical response to metabolic correction.

## 2 Case description

### 2.1 Patient information

A 70-year-old Chinese man was admitted because of right-sided involuntary limb movements lasting one month. He had a 15-year history of type 2 diabetes mellitus and hypertension, both poorly controlled. Diabetes had been managed irregularly with oral hypoglycemic agents and insulin, with the most recent HbA1c of 15.2%. One month earlier, he developed subacute right-predominant dance-like movements of the limbs, accompanied by foot and lower-limb pain and an 11 kg weight loss over the preceding two months. He also had a recent cerebral infarction treated with traditional Chinese medicine.

### 2.2 Clinical findings

Laboratory evaluation confirmed diabetic ketosis and extremely poor long-term glycemic control. Key abnormal laboratory findings are summarized in **Table 1**. Complementary examinations revealed multiple microvascular and macrovascular diabetic complications (**Table 2**). Brain MRI showed only age-related changes without classic striatal T1 hyperintensity (**Figure 1**). Extensive negative work-up for malignancy, autoimmunity, and infection is provided in the **Supplementary Table 1**.

**Table 1:** Laboratory findings.

Category	Parameter	Result	Reference range
Complete blood count	Red blood cell count	3.71 × 10^12^/L	4.0–5.5
	Hemoglobin	114 g/L	130–175
Inflammation	C-reactive protein (CRP)	39.29 mg/L	<10
Urinalysis	Protein	1+	Negative
	Ketones	2+	Negative
	Glucose	3+	Negative
Renal	Urinary microalbumin	119.00 mg/L	<30
	Urine albumin-to-creatinine ratio	113.15 mg/g	<30
Glycemic control	Fasting plasma glucose	7.73 mmol/L	3.9–6.1
	HbA1c	15.2%	4.0–6.0
Arterial blood gas	pH	7.45	7.35–7.45
	Actual bicarbonate (HCO_3_^-^)	29.20 mmol/L	21–28
	Anion gap	-2.2 mmol/L	8–16
Lipid	HDL-cholesterol	0.86 mmol/L	>1.0
Liver function	Total protein	53.4 g/L	65–85
	Albumin	31.1 g/L	40–55
	Prealbumin	107 mg/L	200–400
	AST	56 U/L	15–40
Bone/Mineral	25-hydroxyvitamin D	10.50 ng/mL	30–100
	Serum calcium	1.95 mmol/L	2.1–2.6
Endocrine	Morning cortisol	154.00 nmol/L	171–536
	Estradiol	168.1 pmol/L	28–156
	LH	10.42 mIU/mL	1.7–8.6

**Table 2:** Positive complementary examination findings.

Examination	Positive findings
Fundoscopy	Scattered retinal hemorrhages
Ocular B-ultrasound	Bilateral vitreous opacities with posterior vitreous detachment
Electromyography	Polyneuropathy
Electrocardiogram	Sinus tachycardia
Lower-extremity arterial ultrasound	Bilateral intima-media thickening with plaque formation
Carotid ultrasound	Bilateral intima-media thickening with plaques
Echocardiography	Basal septal hypertrophy, ascending aortic dilatation, mild aortic/mitral/tricuspid regurgitation, left ventricular diastolic dysfunction
Urinary ultrasound	Right renal cyst, mild prostatic enlargement
Thyroid ultrasound	TI-RADS 3 nodule
Brain MRI	Fazekas grade 1 white-matter hyperintensities; left cerebellar low-signal focus (possible old microbleed); no acute basal ganglia lesions or classic T1 hyperintensity

**Figure 1:**
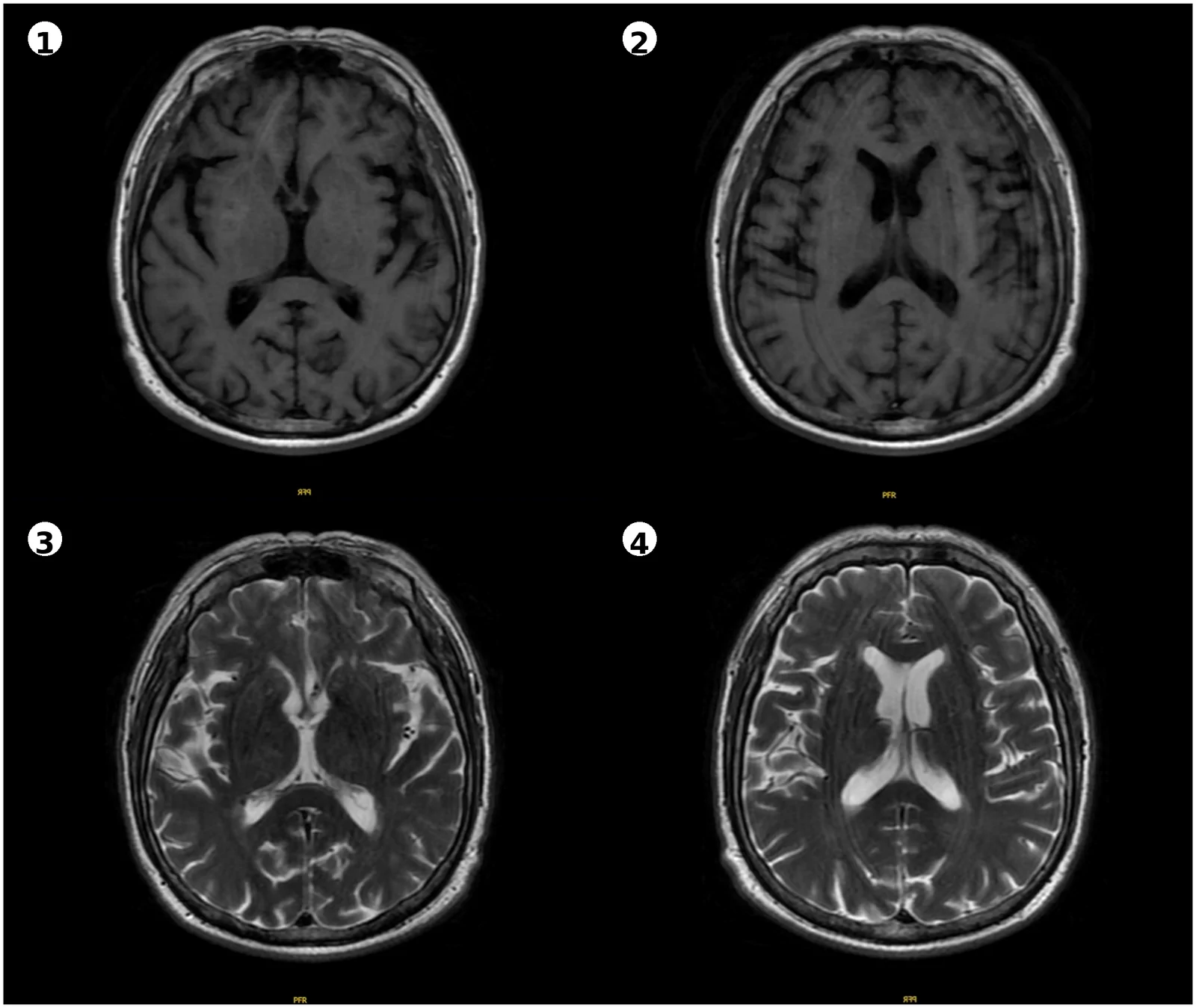
Axial T1-weighted images (1, 2) and T2-weighted images (3, 4) at the level of the basal ganglia and lateral ventricles. No acute basal ganglia lesions or classic T1 hyperintensity of diabetic striatopathy are identified. Mild age-related white-matter changes (Fazekas grade 1) and mild ventriculomegaly are noted.

### 2.3 Diagnostic assessment

Laboratory evaluation confirmed longstanding, extremely poorly controlled type 2 diabetes mellitus (HbA1c 15.2%) with concurrent diabetic ketosis. Arterial blood gas analysis showed pH 7.45, actual bicarbonate 29.2 mmol/L, and a low anion gap (−2.2 mmol/L), confirming the absence of metabolic acidosis and supporting a diagnosis of isolated diabetic ketosis rather than diabetic ketoacidosis. Multiple microvascular (retinopathy, nephropathy, polyneuropathy) and macrovascular (carotid and lower-extremity atherosclerosis, diastolic dysfunction) complications were present. Extensive laboratory and imaging work-up effectively excluded malignancy, autoimmune encephalitis, paraneoplastic syndromes, acute infectious or hemorrhagic causes, and other structural neurological etiologies of the movement disorder. Mild abnormalities in nutritional and inflammatory markers were consistent with chronic metabolic dysregulation and the recent infection of the diabetic foot ulcer.

### 2.4 Therapeutic intervention

Glycemic management consisted of insulin degludec/liraglutide 20 U administered before breakfast, insulin aspart 5 U before each meal, and acarbose 100 mg three times daily. Intravenous fluid resuscitation was provided until ketonuria resolved. Low-dose haloperidol (1 mg twice daily) was added for symptomatic control of the severe involuntary movements and was gradually tapered after clinical improvement.

Additional therapies addressed the patient’s concurrent diabetic complications and included epalrestat and mecobalamin for peripheral neuropathy, beraprost and cilostazol to improve microcirculation, antibiotics together with local wound care for the early diabetic foot infection, antihypertensive agents, a statin, an antiplatelet agent, and vitamin D plus calcium supplementation.

### 2.5 Follow-up and outcomes

Within two weeks the amplitude and frequency of choreiform movements decreased dramatically; right-sided movements became minimal. Fasting glucose stabilized at 5–7 mmol/L and postprandial glucose at 7–9 mmol/L. The patient was discharged on 28 October 2024. Haloperidol was gradually discontinued (**Figure 2**). At three-month follow-up he remained free of choreiform or ballistic movements with improved glycemic control (HbA1c 7.3%). No adverse effects attributable to haloperidol were observed. Adherence to insulin and lifestyle measures was reported as good after discharge education. The patient’s timeline of the episode of care is presented in **Figure 3**.

**Figure 2:**
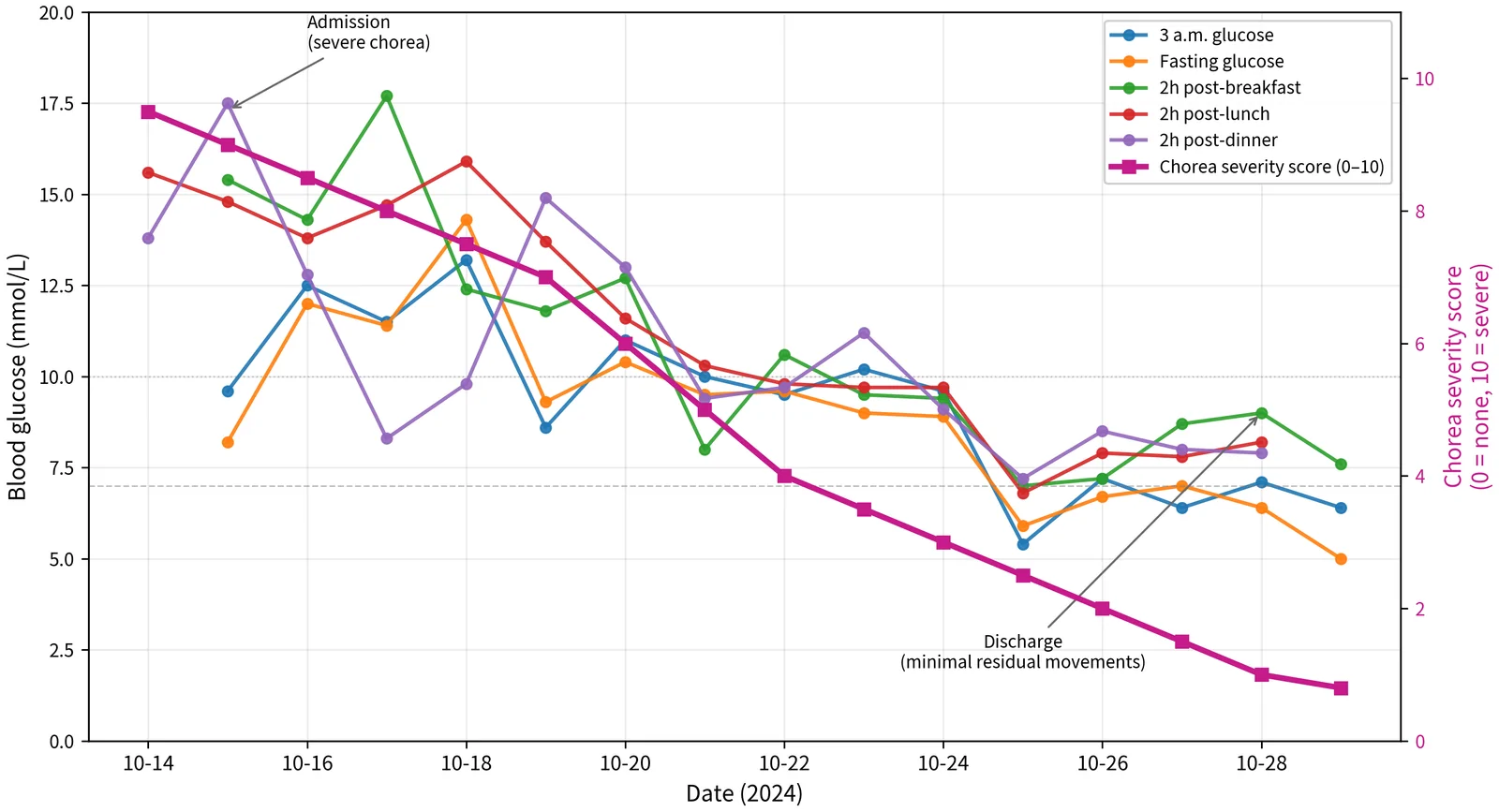
Concurrent improvement of glycemic control and hemichorea-hemiballism during hospitalization. Daily blood glucose values (left y-axis) and clinician-rated chorea severity score (right y-axis; 0 = no involuntary movements, 10 = severe continuous choreiform/ballistic movements). The score is a simple visual analog scale used to illustrate the temporal relationship between metabolic correction and clinical improvement; it is not a validated instrument.

**Figure 3:**
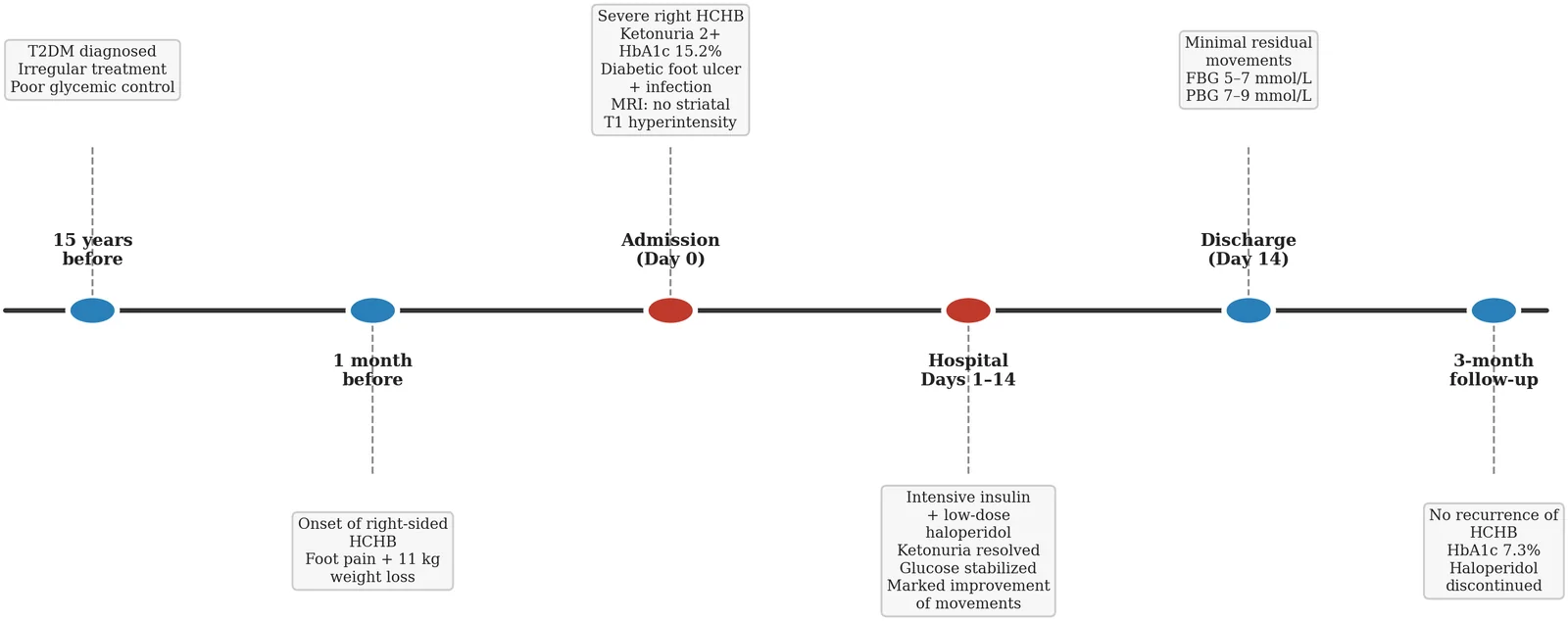
Timeline of the episode of care. Key clinical events and the most important abnormal paraclinical findings from 15 years before admission to the 3-month follow-up. Red markers highlight the admission and intensive treatment period. HCHB, hemichorea-hemiballism; FBG, fasting blood glucose; PBG, postprandial blood glucose.

## 3 Discussion

Diabetic striatopathy (DS), also known as hyperglycemia-associated hemichorea-hemiballism, was first described by Bedwell in 1960 (7). It most commonly affects elderly individuals of Asian descent and is classically linked to non-ketotic hyperglycemia (8). Although most reported cases are non-ketotic, an increasing number of observations confirm that DS can also occur in the presence of ketosis or diabetic ketoacidosis (9, 10). The present case of a 70-year-old man with longstanding, extremely poorly controlled type 2 diabetes mellitus who developed right-predominant hemichorea-hemiballism in the setting of diabetic ketosis adds to this still-limited literature.

Clinically, the involuntary movements in our patient were typical of DS: predominantly unilateral, continuous choreiform and ballistic movements affecting the limbs, worse with activity and absent during sleep (2, 11). This presentation is consistent with the majority of previously reported cases, whether ketotic or non-ketotic.

The pathophysiology of DS remains incompletely understood. Proposed mechanisms include striatal ischemia or hypoperfusion, petechial hemorrhage, metabolic dysfunction of GABAergic and cholinergic neurons secondary to hyperglycemia, dopaminergic hyperactivity, and hyperviscosity-related changes (**Figure 4**) (1, 6, 12–15). In non-ketotic hyperglycemia, depletion of GABA (which is used as an alternative energy substrate) has been suggested as a key factor. However, this explanation is less satisfactory in ketotic states, in which acetoacetate is available. Alternative or complementary mechanisms, such as dopamine receptor hypersensitivity or microvascular ischemia, may therefore play a more prominent role when ketosis is present (16). The rapid clinical improvement after metabolic correction in our patient supports the concept that the movement disorder is largely reversible once the metabolic insult is removed.

**Figure 4:**
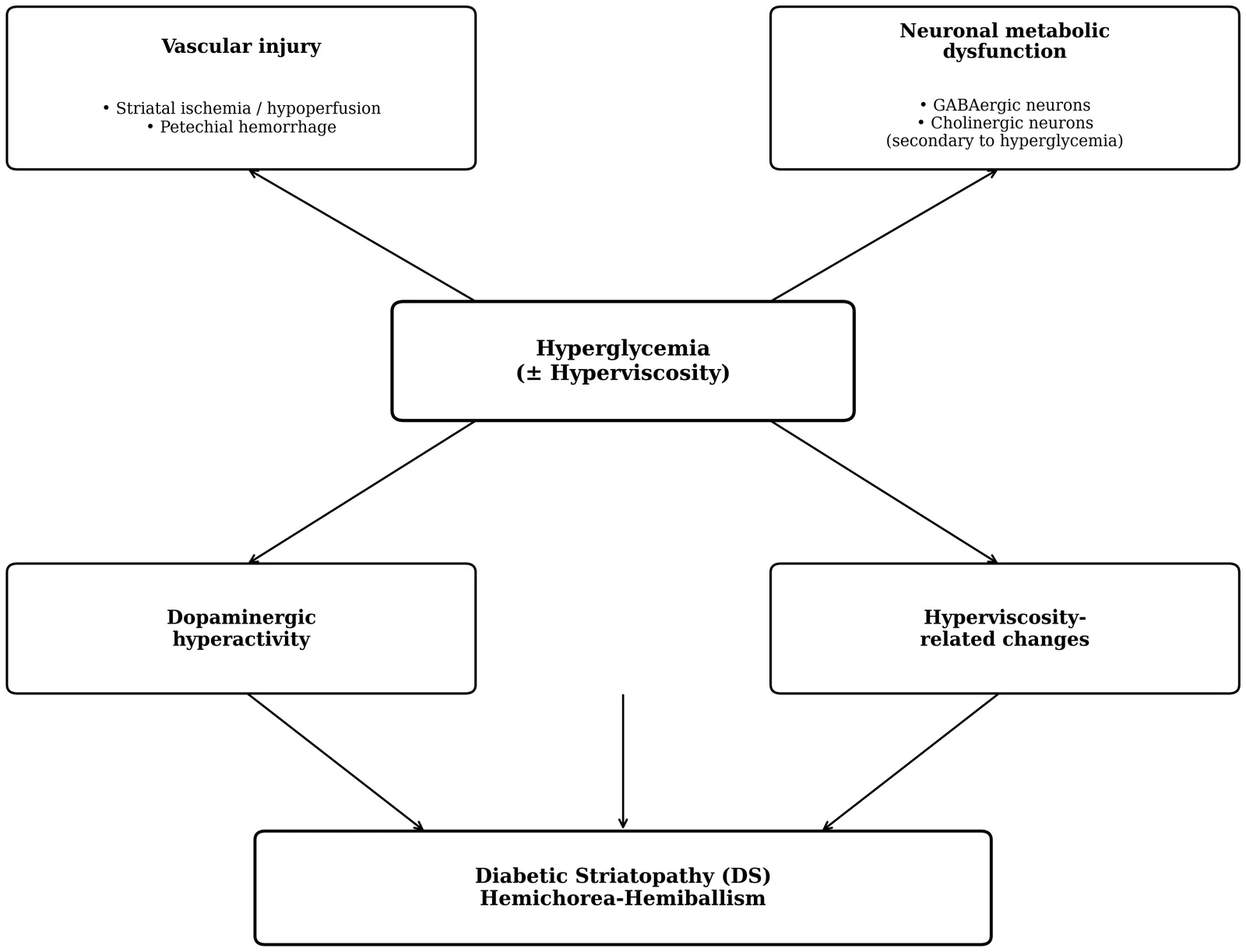
Proposed pathophysiological mechanisms of diabetic striatopathy. Hyperglycemia (± hyperviscosity) may lead to hemichorea-hemiballism through vascular injury, neuronal metabolic dysfunction, dopaminergic hyperactivity, and hyperviscosity-related changes.

A notable feature of the present case is the absence of the classic contralateral basal-ganglia T1 hyperintensity on MRI. While characteristic imaging findings are helpful, they are not invariably present. Approximately 7% of patients with clinically evident hyperglycemic chorea show no striatal abnormalities on CT or MRI, and this presentation has been termed “clinically isolated diabetic striatopathy” (1, 12). Diagnosis therefore rests primarily on the temporal association with severe hyperglycemia and ketosis, exclusion of alternative etiologies, and the clinical response to glycemic control.

Treatment centers on prompt and sustained correction of hyperglycemia and ketosis. Most patients experience substantial improvement within days to weeks. Short-term use of a dopamine-receptor antagonist (haloperidol in this case) can provide symptomatic relief for severe movements and was well tolerated. Long-term anti-chorea medication is usually unnecessary once metabolic control is achieved (1, 9).

Strengths of this report include the detailed documentation of metabolic parameters, the clear temporal relationship between ketosis correction and neurological improvement, and the 3-month follow-up confirming sustained resolution. Limitations include the lack of quantitative serum β-hydroxybutyrate measurement, the absence of serial advanced neuroimaging, and the single-case nature of the observation, which precludes firm conclusions about pathogenesis.

In conclusion, hemichorea-hemiballism can occur in diabetic ketosis as well as non-ketotic hyperglycemia. Classic striatal imaging changes are helpful but not mandatory for diagnosis when the clinical–metabolic correlation is strong, which means hemichorea-hemiballism can occur in association with diabetic ketosis, even in the absence of classic striatal imaging abnormalities. Intensive glycemic control and ketone clearance remain the primary therapy; short-term dopamine-receptor blockade can be used safely for severe symptoms. Early recognition and aggressive metabolic correction remain the cornerstone of management and can lead to excellent neurological recovery. Clinicians should maintain a high index of suspicion for this rare but treatable condition in elderly patients with poorly controlled diabetes who present with acute or subacute unilateral involuntary movements. And long-term adherence education is essential to prevent recurrence in patients with previously chaotic diabetes management.

## 4 Patient perspective

The patient reported that the uncontrollable movements were frightening and severely interfered with daily activities. After blood-glucose stabilization and resolution of the movements he experienced marked relief and improved quality of life. He expressed a strong commitment to better adherence to insulin therapy and regular follow-up.

## Data Availability

The original contributions presented in the study are included in the article/**Supplementary Material**. Further inquiries can be directed to the corresponding author.
