# Supplementary material for: Case Report: Diabetic ketosis-associated hemichorea-hemiballism in an elderly patient with longstanding poorly controlled type 2 diabetes mellitus
Source: Front Endocrinol (Lausanne). 2026 Oct 26;17:1991954. doi: 10.3389/fendo.2026.1991954 (PMC13745212; doi:10.3389/fendo.2026.1991954)
Supplement: Supplementary file 1 [file Table1.docx]

Supplementary Table 1. Additional laboratory screening results

| **Category** | **Parameter** | **Result** | **Reference range** |
| --- | --- | --- | --- |
| Tumor markers | Ferritin | 280.50 ng/mL | 30–400 |
|  | Neuron-specific enolase (NSE) | 14.09 ng/mL | <16.3 |
|  | Cytokeratin 19 fragment | 2.83 ng/mL | <3.3 |
|  | Pro-gastrin-releasing peptide | 34.87 pg/mL | <50 |
|  | Prostatic acid phosphatase | 0.98 ng/mL | <3.5 |
|  | Total PSA | 0.386 ng/mL | <4.0 |
|  | Free PSA | 0.169 ng/mL | — |
|  | Free-to-total PSA ratio | 0.438 | — |
|  | Squamous cell carcinoma antigen | 1.87 ng/mL | <1.5 |
|  | CA153 | 6.990 U/mL | <25 |
|  | CA724 | 0.70 U/mL | <6.9 |
|  | CA125 | 8.300 U/mL | <35 |
|  | CA199 | 9.990 U/mL | <37 |
|  | AFP | 1.580 ng/mL | <7.0 |
|  | CEA | 2.020 ng/mL | <5.0 |
| Rheumatologic panel | IgE | 110.20 IU/mL | — |
|  | ENA, ANA, ANCA, IgG4 | Negative / unremarkable | — |
| Other | Urea, creatinine, electrolytes, uric acid, magnesium, phosphorus | Within normal limits | — |
|  | Coagulation profile | Within normal limits | — |
|  | Infectious serology | Within normal limits | — |
